# Modelling the evolution of HIV‐1 virulence in response to imperfect therapy and prophylaxis

**DOI:** 10.1111/eva.12458

**Published:** 2017-02-11

**Authors:** David R. M. Smith, Nicole Mideo

**Affiliations:** ^1^ Department of Ecology and Evolutionary BiologyUniversity of TorontoTorontoONCanada

**Keywords:** adaptive dynamics, antiretroviral therapy, pre‐exposure prophylaxis, set‐point viral load, virulence evolution

## Abstract

Average HIV‐1 virulence appears to have evolved in different directions in different host populations since antiretroviral therapy first became available, and models predict that HIV drugs can select for either higher or lower virulence, depending on how treatment is administered. However, HIV virulence evolution in response to “leaky” therapy (treatment that imperfectly suppresses viral replication) and the use of preventive drugs (pre‐exposure prophylaxis) has not been explored. Using adaptive dynamics, we show that higher virulence can evolve when antiretroviral therapy is imperfectly effective and that this evolution erodes some of the long‐term clinical and epidemiological benefits of HIV treatment. The introduction of pre‐exposure prophylaxis greatly reduces infection prevalence, but can further amplify virulence evolution when it, too, is leaky. Increasing the uptake rate of these imperfect interventions increases selection for higher virulence and can lead to counterintuitive increases in infection prevalence in some scenarios. Although populations almost always fare better with access to interventions than without, untreated individuals could experience particularly poor clinical outcomes when virulence evolves. These findings predict that antiretroviral drugs may have underappreciated evolutionary consequences, but that maximizing drug efficacy can prevent this evolutionary response. We suggest that HIV virulence evolution should be closely monitored as access to interventions continues to improve.

## 1 Introduction

The evolution of parasites in response to human interventions is a fundamental challenge to public health. A growing number of parasites have evolved means of resisting chemotherapeutic drugs and vaccines, limiting or altogether eliminating options to prevent and treat the diseases they cause (reviewed in Gandon & Day, 2008; Bell, Schellevis, Stobberingh, Goossens, & Pringle, 2014). In addition to conventional resistance mechanisms (e.g., efflux pumps to thwart drugs or antigenic variation to escape vaccine‐induced immunity), experiments have shown that parasite virulence can evolve in response to—and mitigate the effects of—medical interventions, as exemplified by Marek's disease virus in response to vaccines (Read et al., 2015), and rodent malaria parasites in response to drugs (Schneider et al., 2012) and vaccines (Barclay et al., 2012). The extent of this kind of evolution in nonexperimental systems is poorly understood, but there is some evidence of vaccine‐driven virulence evolution in parasites of humans (pertussis, Mooi et al., 2009), cats (feline calicivirus, Radford, Dawson, Coyne, Porter, & Gaskell, 2006) and poultry (Marek's disease virus, Nair, 2005; avian infectious bursal disease virus, van den Berg, 2000). Through the development of general theory, Gandon, Mackinnon, Nee, and Read (2001) formalized the prediction that imperfectly effective, or “leaky” vaccines can drive virulence evolution. Importantly, this study showed that the strength and even the direction of selection can change depending on the precise vaccine target (e.g., parasite proteins or pathways related to growth, infection or transmission) and the subtleties of any trade‐offs between virulence and other disease traits (e.g., transmission). Virulence may thus be expected to evolve idiosyncratically in response to different interventions in different host–parasite systems.

The quantitative relationship between virulence and transmission in a human disease system has arguably been most thoroughly studied in HIV‐1 (reviewed in Fraser et al., 2014). In HIV infections, viral load refers to the density of virus in the blood stream (virions/ml of blood plasma). Viral load typically spikes during primary HIV infection, when the virus first establishes itself in host CD4^+^ cells, and towards the end of infection, when CD4^+^ cell concentrations crash and hosts progress to AIDS. However, during the lengthy asymptomatic phase of infection, which can last from 2 to over 20 years, viral load fluctuates about a steady value (Babiker, Darby, Angelis, Ewart, & Porter, 2000). This so‐called set‐point viral load (SPVL) varies by orders of magnitude between hosts (de Wolf et al., 1997) and underlies a trade‐off between virulence and transmission: hosts with higher SPVL progress to AIDS and death more quickly (Mellors et al., 1997), but are more infectious than those with low SPVL (Quinn et al., 2000). As a result, intermediate SPVL is predicted to maximize average lifetime HIV‐1 transmission (Fraser, Hollingsworth, Chapman, de Wolf, & Hanage, 2007). Importantly, as SPVL is heritable between infections and is in part determined by viral genes (Alizon et al., 2010; Fraser et al., 2014), intermediate SPVL and, hence, intermediate virulence are believed to have evolved over the course of the HIV‐1 pandemic (Fraser et al., 2007; Herbeck, Mittler, Gottlieb, & Mullins, 2014; Lythgoe, Pellis, & Fraser, 2013; Shirreff, Pellis, Laeyendecker, & Fraser, 2011).

This theoretically optimal intermediate SPVL has been characterized for populations without access to HIV interventions, but antiretroviral therapy (ART) is now widely used to suppress viral replication in infected hosts. Although these drugs have been circulating in various incarnations for decades (Vella, Schwartländer, Sow, Eholie, & Murphy, 2012), their effect on HIV‐1 virulence evolution remains contested. Two recent modelling studies have predicted that ART selects for lower SPVL when more virulent infections are more likely to be treated (Payne et al., 2014; Roberts, Goulder, & Mclean, 2015). However, the WHO's “test and treat” policy now recommends the immediate initiation of ART upon HIV diagnosis, regardless of prognostic indicators such as viral load and CD4^+^ cell count (WHO, 2015), which curtails this proposed mechanism of selection in populations adhering to such a policy. In contrast, it has been suggested that treatment could select for higher SPVL by reducing the associated costs of higher virulence (Fraser et al., 2014; Porco, Lloyd‐Smith, Gross, & Galvani, 2005). Using stochastic, individual‐based simulations, Herbeck et al. (2016) show that increasing the coverage of ART selects for higher SPVL when all infections are equally likely to be treated. Although the predictions of these models are conflicting, the available data are also conflicting. Average SPVL has increased in some populations and decreased in others since the introduction of ART (Herbeck et al., 2012). As different populations are likely to experience different selection pressures, understanding the role and relative influence of those potential pressures is important.

A key assumption underlying past models of HIV‐1 virulence evolution is that treated hosts are unable to transmit their infections. Although it is true that ART greatly reduces transmission risk, data demonstrate that treated hosts do transmit (Anglemyer et al., 2013; Baggaley, White, Hollingsworth, & Boily, 2013; Ratmann et al., 2016), and any viruses that are able to transmit when exposed to ART will have an evolutionary advantage in highly treated host populations. Treatment with ART is thus analogous to the use of imperfect antigrowth vaccines, which are predicted to select for higher virulence (Gandon et al., 2001). Additionally, past models have not considered that antiretroviral drugs are now also used for prevention. Pre‐exposure prophylaxis (PrEP) is a nascent HIV prevention strategy whereby uninfected hosts take drugs that are similar to (or the same as) ART in order to reduce their susceptibility to infection (WHO, 2015). PrEP thus superficially resembles anti‐infection vaccines, which are predicted to have no effect on virulence evolution on their own, or may select for lower virulence under certain conditions (e.g., with superinfection; Gandon et al., 2001). However, PrEP differs from traditional anti‐infection vaccines in an important way: if a host on PrEP becomes infected, the viruses they harbour will immediately be exposed to antiretroviral drugs. For this reason, PrEP may be expected to increase the strength of selection in response to ART. But to what extent this is true and how this effect balances with the epidemiological benefits of prevention are hard to predict.

Here, we develop a compartmental model of HIV‐1 transmission to examine the epidemiological impacts of interventions, and we use adaptive dynamics to explore the phenotypic evolution of virulence in response. We first predict the trajectory and endpoint of SPVL evolution in the face of ART under a test and treat policy and under the assumption that treated hosts remain able to transmit at a reduced rate. We then introduce PrEP and examine the evolutionary consequences of increasing the availability of these prophylactic drugs, as is being encouraged by the WHO and other public health agencies (WHO, 2015). Finally, we examine the net epidemiological and clinical effects of interventions when SPVL evolves.

## 2 Methods

### 2.1 Model

We developed a compartmental model of frequency‐dependent HIV transmission to explore the epidemiological and evolutionary consequences of ART and PrEP. This system of ordinary differential equations (ODEs) tracks the frequencies of four host types in a population of sexually active adults: susceptible (*S*), susceptible on PrEP (*P*), infected (*I*) and infected on ART (*T*):

(1) $$\frac{dS}{dt}=\theta -\left(\beta_{I}I+\beta_{T}T\right)S-\left(\mu +f_{P}\right)S$$

(2) $$\frac{dP}{dt}=f_{P}S-\eta_{P}\left(\beta_{I}I+\beta_{T}T\right)P-\mu P$$

(3) $$\frac{dI}{dt}=\left(\beta_{I}I+\beta_{T}T\right)S-\left(\mu +\alpha_{I}+f_{T}\right)I$$

(4) $$\frac{dT}{dt}=f_{T}I+\eta_{P}\left(\beta_{I}I+\beta_{T}T\right)P-\left(\mu +\alpha_{T}\right)T$$

where θ is the rate at which individuals enter the sexually active population (and functions to maintain a constant population size); β_*I*_ and β_*T*_ are the per‐capita rates of HIV‐1 transmission from untreated and treated infected hosts, respectively; α_*I*_ and α_*T*_ are the rates of progression to AIDS in untreated and treated infected hosts; μ is the rate of background mortality; η_*P*_ is a coefficient that reduces the susceptibility of hosts on PrEP; and *f*_*T*_ and *f*_*P*_ are rates of ART and PrEP uptake (see full list of parameters in Table 1). For individuals in the infected class, the ART uptake rate reflects a test and treat policy—all individuals, regardless of SPVL, are equally likely to take up treatment in a given unit of time, and the inverse of the uptake rate describes the average duration of infection prior to starting treatment. All transmission is assumed to occur during the asymptomatic phase of infection, when SPVL is expressed and the majority of HIV‐1 transmission occurs (Bellan, Dushoff, Galvani, & Meyers, 2015; Hollingsworth, Anderson, & Fraser, 2008; Powers et al., 2011). Excluding primary HIV infection and AIDS allows us to subsume within‐host processes into between‐host vital rates. Treated hosts are assumed to have reduced SPVL and hence reduced rates of transmission and progression to AIDS (i.e., β_*T*_ **<* *β_*I*_; α_*T*_ **<* *α_*I*_). Hosts on PrEP move directly into the treated class upon infection, because (a) hosts undergo regular HIV testing when taking PrEP (WHO, 2015) and (b) hosts on PrEP are assumed to continue taking their medication if unknowingly infected, and the continued use of PrEP postinfection is likely to behave functionally like ART, as similar drugs are used (García‐Lerma et al., 2008; Prada et al., 2008).

**Table 1:** Default variable and parameter values of the model

Symbol	Description (units)	Default value or range
*S*	Frequency of susceptible hosts	0–1
*P*	Frequency of susceptible hosts on PrEP	0–1
*I*	Frequency of infected hosts	0–1
*T*	Frequency of infected hosts on ART	0–1
*V*	SPVL (virions/ml blood plasma)	38,465.1
α_*I*_	Rate of progression to AIDS (year^−1^)	0.151
β_*I*_	HIV transmission rate (year^−1^)	0.234
μ	Background mortality rate (year^−1^)	0.02
*f*_*T*_	ART uptake rate (year^−1^)	0–1
*f*_*P*_	PrEP uptake rate (year^−1^)	0–1
*r*_*T*_	ART efficacy (log_10_ reductions in SPVL) [low, medium, high]	1, 1.5, 2
*r*_*P*_	PrEP efficacy [low, medium, high]	0.2, 0.5, 0.8
*η*_*P*_	Susceptibility on PrEP	1 − *r*_*P*_
θ	Population input rate (year^−1^)	μ + α_*I*_ *I* + α_*T*_ *T*
*D*_max_	Maximum duration of asymptomatic infection (years)	25.4^a^
*D*_50_	Viral load at which *D* is half maximum	3,058^a^
*D*_*k*_	Duration Hill coefficient	0.41^a^
β_max_	Maximum infection rate (year^−1^)	0.317^a^
β_50_	Viral load at which β is half maximum	13,938^a^
β_k_	Transmission rate Hill coefficient	1.02^a^

a Parameter values obtained from Fraser et al. (2007).

In our model, rates of transmission and disease progression are governed by the SPVL trade‐off quantified by Fraser et al. (2007), who described the relationship between SPVL (*V*) and the duration of untreated asymptomatic infection (*D*_*I*_) using a decreasing Hill function,

(5) $$D_{I}\left[V\right]=\frac{D_{\max}D_{50}^{D_{k}}}{V^{D_{k}}+D_{50}^{D_{k}}}$$

where *D*_max_ is the maximum duration of infection, *D*_50_ is the viral load at which duration is half its maximum, and *D*_*k*_ is a rate‐determining constant. Given *D*_*I*_[*V*] as the average duration of untreated asymptomatic infection, and assuming exponentially distributed wait times, then the rate of progression from asymptomatic infection to AIDS (α_*I*_) is represented by the inverse of Equation (5),

(6) $$\alpha_{I}\left[V\right]=\frac{V^{D_{k}}+D_{50}^{D_{k}}}{D_{\max}D_{50}^{D_{k}}}.$$

Fraser et al. (2007) also described a saturating relationship between HIV‐1 transmission rate and SPVL using an increasing Hill function,

(7) $$\beta_{I}\left[V\right]=\frac{\beta_{\max}V^{\beta_{k}}}{V^{\beta_{k}}+\beta_{50}^{\beta_{k}}}$$

where β_max_ is the maximum rate of transmission from an infected host, β_50_ is the viral load at which the rate of transmission is half its maximum, and β_*k*_ is a rate‐determining constant. The product of Equations (5) and (7) is HIV‐1 transmission potential, the expected number of transmission events from a single infected host over the full duration of asymptomatic infection (Fraser et al., 2007). This formulation assumes that transmission ceases with progression to AIDS, as assumed in other HIV virulence evolution models (Blanquart et al., 2016; Payne et al., 2014; Roberts et al., 2015). According to these functions, and the parameter estimates listed in Table 1, transmission is maximized when *V *= 10^4.52^. This estimate is broadly consistent with the mean of SPVL distributions observed in human populations prior to the rollout of antiretroviral drugs (Fraser et al., 2007).

We modified the paradigm set forth by Gandon et al. (2001) to model how PrEP affects host susceptibility, and how ART affects rates of transmission and disease progression through a reduction in within‐host viral load. Specifically,

(8) $$\eta_{P}=\left(1-r_{P}\right)$$

(9) $$V_{T}=\frac{V}{10^{r_{T}}}$$

(10) $$\alpha_{T}=\alpha_{I}\left[V_{T}\right]$$

(11) $$\beta_{T}=\beta_{I}\left[V_{T}\right]$$

where *r*_*P*_ and *r*_*T*_ represent the efficacies of PrEP and ART, respectively, and *V*_*T*_ represents average viral load in a treated infection. If *r*_*P*_ **= 1, a host using PrEP cannot be infected, while PrEP has no effect if *r*_*P*_ = 0. The interpretation of *r*_*T*_ is slightly different as it captures a log_10_ reduction in viral load due to treatment (i.e., *r*_*T*_ = 2 is a 2 − log_10_ reduction in *V* due to treatment). At the extremes, ART is perfectly effective and eliminates viral load in an infection when *r*_*T*_ **= ∞, and is completely ineffective when *r*_*T*_ = 0.

### 2.2 Parameter estimation

The parameters of this model are likely highly variable in and among different host populations, and we therefore consider broad ranges where numerical predictions are made. However, some parameters can be inferred from the literature. Background mortality is set at μ* *= 0.02, which amounts to an average uninfected host lifespan of 50 years and is consistent with other HIV models (Lythgoe et al., 2013; Roberts et al., 2015). Generally speaking, ART is highly effective, and approximately three quarters of hosts undergoing ART are virologically suppressed (i.e., viral load is below 400 virions/ml; UNAIDS, 2014), but very few studies have estimated the average degree of viral suppression over the full course of treated HIV infection. Cole et al. (2010) recently developed viral copy years (VCY) as a means of measuring cumulative viral burden. Using this metric, mean viral load in an Australian cohort undergoing ART over the course of ten years was estimated to be 10^3.3^ (Wright et al., 2014). However, mean baseline SPVL was not published in this study and ranges from 10^3.5^ to over 10^5^ in different populations (Dorrucci, Rezza, Porter, & Phillips, 2007; Fraser et al., 2007; Gras et al., 2009; Mellors et al., 1996; Müller et al., 2009; Pilcher et al., 2007), so an exact measure of ART efficacy in this cohort cannot be inferred. We also note that individuals in marginalized and low‐income populations are more likely to have reduced or inconsistent adherence to ART due to social, economic and psychological barriers (Boyer et al., 2011) and may be less likely to be represented in longitudinal studies of ART efficacy. Given the limited information available, we arbitrarily set low, medium and high estimates of ART efficacy to *r*_*T*_ **= 1, 1.5 and 2, respectively, which are broadly consistent with other models (Conway & Perelson, 2016).

The uptake rate of ART is also poorly characterized. Globally, 40% of people living with HIV are estimated to be on some form of treatment (UNAIDS, 2014), and from this we infer that the average rate of ART uptake is relatively low. (In our model, 40% of infections are treated at equilibrium when *f*_*T*_ *≈ *0.05, 0.06 and 0.07, for high, medium and low efficacy ART, respectively.) However, access to treatment continues to improve worldwide in pursuit of WHO's goal to treat 90% of all infections (WHO, 2015). Therefore, to study a diversity of treatment scenarios, we consider the consequences of ART over the range 0 ≤ *f*_*T*_ ≤ 1 (where *f*_*T*_ = 1 indicates that 63% of all infected hosts initiate treatment during the first year of infection).

The efficacy of PrEP may exceed 99% in ideal conditions and given perfect drug adherence (Anderson et al., 2012), but efficacy measures from clinical trials range widely. Excluding two trials discontinued due to nonefficacy, PrEP has been found to reduce the risk of HIV acquisition by 39%–86% (McCormack et al., 2015; van der Straten, Van Damme, Haberer, & Bangsberg, 2012), and a recent meta‐analysis found that PrEP reduces HIV risk by 51% compared to placebo (Fonner et al., 2016). Further, as PrEP efficacy hinges vitally on drug adherence (McCormack et al., 2015; van der Straten et al., 2012), the efficacies reported in clinical trials may be biased due to inflated access to drugs and medical care. We therefore consider low, medium and high PrEP efficacy estimates of 0.2, 0.5 and 0.8, respectively. Finally, given considerable uncertainty in the expected rollout of PrEP, we consider the range of PrEP uptake 0 < *f*_*P*_ < 0.1. Recalling that these are annual rates in long‐lived hosts, the upper rate *f*_*P*_ = 0.1 indicates that approximately 10% of all uninfected individuals initiate PrEP within the first year of entering the population.

### 2.3 Analysis

We used an equilibrium analysis to predict how, in the absence of evolution, interventions affect the prevalence of infection in the host population. We derived analytical expressions for equilibria in certain cases, for example, in populations where ART or PrEP are used alone (Appendix S1). One inference from this analysis is that for perfectly effective treatments, and all else being equal, preventing infection in susceptible hosts is a more effective way to control the spread of the virus than preventing transmission from infected individuals (compare S1.13 with S1.18, when *r*_*P*_ = 1 and *V*_*T*_ = 0). When equilibria were not analytically solvable, for example, when imperfect PrEP and ART are used in combination, we used numerical simulations to predict equilibrium infection prevalence and host frequencies. All endemic equilibria reported were found to be locally asymptotically stable in the absence of evolution.

We used an evolutionary invasion analysis to explore the consequences of different intervention scenarios on the between‐host evolution of HIV virulence. This method optimizes invasion fitness, *R*_*m*_, a measure of the growth rate of a rare mutant parasite introduced into a host population where a resident parasite (with a different trait value) is endemic and where the system is at epidemiological equilibrium (Dieckmann, Metz, Sabelis, & Sigmund, 2002; Otto & Day, 2008). When introduced, any mutant parasite that grows in abundance or density (*R*_*m*_ > 0) is assumed to outcompete and replace the resident “strain.” When a resident is at equilibrium and *R*_*m*_ < 0 for all possible mutant trait values, the resident trait is evolutionarily stable and cannot be invaded by any mutant. All else being equal, this is the trait value that should evolve.

Specifically, we used methods of invasion analysis adapted from next‐generation theory (Hurford, Cownden, & Day, 2010; Van Den Driessche & Watmough, 2002) to compute *R*_*m*_. First, we expanded our system of ODEs to include hosts infected with a mutant virus, characterized by a different SPVL:

(12) $$\frac{dS}{dt}=\theta -\left(\beta_{I}I+\beta_{T}T+\beta_{I}^{\prime }I^{\prime }+\beta_{T}^{\prime }T^{\prime }\right)S-\left(\mu +f_{P}\right)S$$

(13) $$\frac{dP}{dt}=f_{P}S-\eta_{P}\left(\beta_{I}I+\beta_{T}T+\beta_{I}^{\prime }I^{\prime }+\beta_{T}^{\prime }T^{\prime }\right)P-\mu P$$

(14) $$\frac{dI}{dt}=\left(\beta_{I}I+\beta_{T}T\right)S-\left(\mu +\alpha_{I}+f_{T}\right)I$$

(15) $$\frac{dT}{dt}=f_{T}I+\eta_{P}\left(\beta_{I}I+\beta_{T}T\right)P-\left(\mu +\alpha_{T}\right)T$$

(16) $$\frac{dI^{\prime }}{dt}=\left(\beta_{I}^{\prime }I^{\prime }+\beta_{T}^{\prime }T^{\prime }\right)S-\left(\mu +\alpha_{I}^{\prime }+f_{T}\right)I^{\prime }$$

(17) $$\frac{dT^{\prime }}{dt}=f_{T}I^{\prime }+\eta_{P}\left(\beta_{I}^{\prime }I^{\prime }+\beta_{T}^{\prime }T^{\prime }\right)P-\left(\mu +\alpha_{T}^{\prime }\right)T$$

where primes denote host classes and infection parameters associated with the mutant. Mutant ODEs can be expressed as their component parts,

(18) $$\frac{d\vec{x}}{dt}=A\vec{x}$$

where $\vec{x}$ is a vector of host classes infected with the mutant virus, and **A** is a nonsingular invasion matrix describing the infection dynamics of the mutant. These terms expand to

(19) $$\frac{d}{dx}\left(\begin{array}{c} I^{\prime }\\ T^{\prime } \end{array}\right)=\left(\begin{array}{cc} \beta_{I}^{\prime }\hat{S}-\left(\mu +\alpha_{I}^{\prime }+f_{T}\right) & \beta_{T}^{\prime }\hat{S}\\ f_{T}+\eta_{P}\beta_{I}^{\prime }\hat{P} & \eta_{P}\beta_{T}^{\prime }\hat{P}-\left(\mu +\alpha_{T}^{\prime }\right) \end{array}\right)\left(\begin{array}{c} I^{\prime }\\ T^{\prime } \end{array}\right)$$

and as a negligible proportion of hosts are infected by the rare mutant, hosts are assumed to be at the stable endemic equilibrium set by the resident virus. The mutant invasion dynamics **A** can then be decomposed as **A = F −** *V*, where

(20) $$F=\left(\begin{array}{cc} \beta_{I}^{\prime }\hat{S} & \beta_{T}^{\prime }\hat{S}\\ \eta_{P}\beta_{I}^{\prime }\hat{P} & \eta_{P}\beta_{T}^{\prime }\hat{P} \end{array}\right)$$

and

(21) $$V=\left(\begin{array}{cc} \mu +\alpha_{I}^{\prime }+f_{T} & 0\\ -f_{T} & \mu +\alpha_{T}^{\prime } \end{array}\right).$$

**F** is a transmission matrix that describes that rate at which existing mutant infections generate new ones, and **V** is a transition matrix that describes the rate at which mutant infections move among and out of infected host classes. Therefore, **V** *^−1^* describes the duration of time that infected and treated hosts are asymptomatically infected with mutant virus. The matrices **F** and **V** *^−1^* satisfy the conditions of the Next‐Generation Theorem (Hurford et al., 2010), where **NGM = FV** *^−1^* is the next‐generation matrix, the elements of which represent the average transmission of mutant virus from each host type. In our model,

(22) $$\mathrm{NGM}=\left(\begin{array}{cc} \frac{\left(\beta_{I}^{{}^{\prime }}+\frac{f_{T}\beta_{T}^{{}^{\prime }}}{\mu +\alpha_{T}^{{}^{\prime }}}\right)\hat{S}}{\mu +\alpha_{I}^{{}^{\prime }}+f_{T}} & \frac{\beta_{T}^{\prime }\hat{S}}{\mu +\alpha_{T}^{{}^{\prime }}}\\ \frac{\eta_{P}\left(\beta_{I}^{{}^{\prime }}+\frac{f_{T}\beta_{T}^{{}^{\prime }}}{\mu +\alpha_{T}^{\prime }}\right)\hat{P}}{\mu +\alpha_{I}^{\prime }+f_{T}} & \frac{\eta_{P}\beta_{T}^{\prime }\hat{P}}{\mu +\alpha_{T}^{{}^{\prime }}} \end{array}\right).$$

The invasion fitness of the mutant virus is calculated from the spectral radius of the **NGM** (ρ), which approximates the total lifetime number of transmission events from a host infected with a rare mutant. Therefore, a mutant virus invades the host population when ρ(**NGM**) > 1. In this way, ρ(**NGM**) is analogous to the basic reproduction ratio of infection, *R*_0_ (Hurford et al., 2010), and so, conventionally, a mutant invades when its reproduction ratio *R*_0_ > 1.

The value of SPVL that maximizes *R*_0_ is the value that maximizes between‐host viral fitness. We find this by evaluating the selection gradient of *R*_0_ with respect to mutant SPVL, *V*′:

(23) $${\left.\frac{\partial R_{0}}{\partial V^{\prime }}\right|}_{V^{\prime }=V}$$

which amounts to a linear approximation of the derivative of invasion fitness taken about the resident trait value. A positive (negative) slope indicates that higher (lower) mutant trait values confer higher fitness than the resident trait.

The root of the selection gradient therefore describes a fitness maximum when

(24) $${\left.\frac{\partial^{2}R_{0}}{\partial {V^{\prime }}^{2}}\right|}_{V^{\prime }=V}<0.$$

A fitness maximum thus represents the “optimal” parasite strategy, but this trait is only predicted to evolve when it is evolutionarily stable, meaning that it reaches stable endemic equilibrium when introduced into the population. SPVL is evolutionarily stable (denoted V*) when

(25) $${\left.\frac{\partial }{\partial V}\left({\left.\frac{\partial R_{0}}{\partial V^{\prime }}\right|}_{V^{\prime }=V}\right)\right|}_{V=V^{*}}<0$$

meaning that higher values of *V*′ invade when *V* < V* and lower values invade when *V* > V*, and hence, trait values converge towards V* over successive invasions (Otto & Day, 2008). We note throughout whether “optimal” values of SPVL are evolutionarily stable, and show a numerical example of convergence stability calculations in Appendix S3. Mutant invasion conditions were derived analytically and interpreted where possible in Appendix S2.

Finally, we estimated the potential public health consequences of intervention‐driven virulence evolution. To do this, we reran our equilibrium analyses in different intervention scenarios, but seeded host populations with viruses with evolutionarily stable SPVL. We then compared infection prevalence and the average duration of HIV infection in epidemics with evolved and unevolved SPVL. This allowed us to broadly assess the net clinical and epidemiological effects of ART and PrEP when they cause virulence to evolve.

## 3 Results

**Figure 1:**
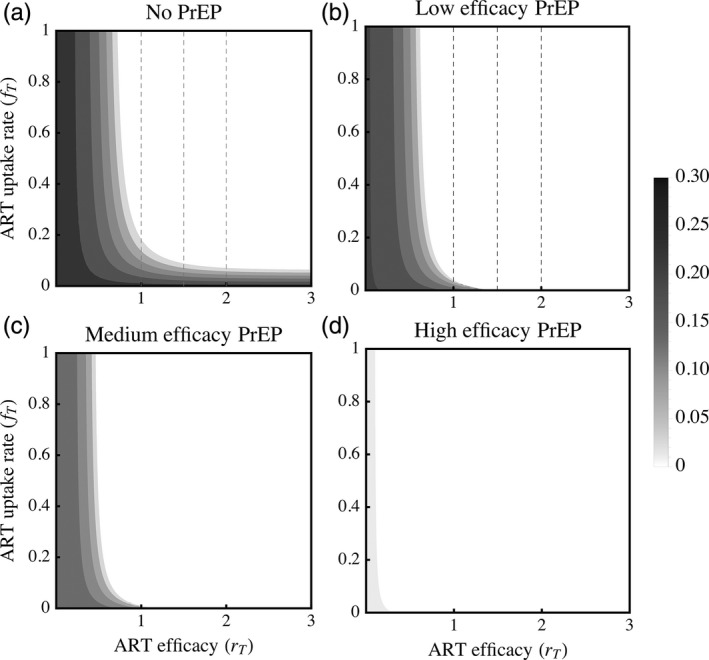
In the absence of evolution, the equilibrium prevalence of HIV infection ($\hat{I}+\hat{T}$) decreases (lighter grey) with both the efficacy and uptake rate of interventions. White regions indicate that drugs have eradicated HIV from the host population, hence endemic HIV infection does not persist over the bulk of ART parameter space explored here. (a) In the absence of PrEP, low efficacy drugs (*r*_*T*_ = 1) eradicate HIV when the uptake rate *f*_*T*_ > 0.2 (i.e., average time to ART initiation is less than five years). When low (b; *r*_*P*_ = 0.2) or medium (c; *r*_*P*_ = 0.5) efficacy PrEP is used in conjunction with ART, eradication can occur in the absence of ART uptake, because those on PrEP are assumed to be treated when infected. (d) With highly effective PrEP (*r*_*P*_ = 0.8), endemic HIV infection can only persist if ART is very ineffective. All plots with PrEP assume uptake rate *r*_*P*_ = 0.01, indicating that approximately 18% of hosts are on PrEP within 20 years of entering the population. Vertical dashed lines (left to right, *r*_*T*_ **= 1, 1.5, 2) correspond, respectively, with the low, medium and high estimates of ART efficacy in Figure 4

In the absence of viral evolution, ART has the expected effect of reducing the equilibrium prevalence of HIV infection (Figure 1a). Given default parameter estimates (Table 1), approximately 27% of hosts become infected in our model population when no treatment options are available, but increases in either the efficacy of ART (*r*_*T*_) or its rate of uptake (*f*_*T*_) contribute to sharp declines in prevalence. When drugs are highly effective, their introduction into the host population eliminates endemic HIV infection (white regions in Figure 1), even at relatively low uptake rates. Above moderate rates of ART uptake (*f*_*T*_ > 0.2), further increases do not have a substantial effect on infection prevalence, regardless of drug efficacy, as most infections are already treated at equilibrium. Unsurprisingly, combining PrEP and ART offers additional epidemiological benefits (Figure 1, b–d). Note that this is true even if PrEP is completely ineffective (*r*_*P*_ = 0), as increasing the rate of PrEP uptake ultimately leads to more infections being treated. In Appendix S4.5, we estimate how long it takes for interventions to eradicate HIV transmission, assuming no viral evolution. As expected, more effective and more highly used drugs lead to more rapid eradication, but even highly effective and common treatments take decades if not centuries to eliminate transmission.

**Figure 2:**
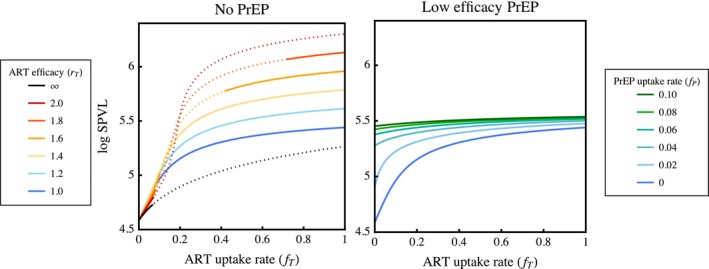
Higher SPVL evolves (solid lines) as the rate of ART uptake increases. Left: when ART is perfectly effective (black line), there is an increase in the level of SPVL that maximizes transmission, but these values are mostly evolutionarily unstable (dotted lines) and are thus not expected to be maintained in the population. However, high SPVL tends to evolve when ART is leaky (coloured lines), and more effective treatment entails higher levels of evolved SPVL (cool to warm colours indicate increasing ART efficacy). When ART is highly effective, higher SPVL only evolves when drugs have a high uptake rate, because these more virulent strains transmit best in well‐treated populations. Right: increasing the uptake rate of leaky PrEP leads to the evolution of higher SPVL. Here, we assume low efficacy interventions (*r*_*T*_ = 1, *r*_*P*_ = 0.2), because HIV is generally evolutionarily unstable when PrEP is highly effective

The use of leaky ART imperfectly suppresses viral load, reducing but not eliminating transmission and disease progression in treated hosts. This generates selection for the compensatory evolution of higher SPVL, as infections that retain higher viral loads when treated are more likely to transmit. We show in Figure 2 how increasing the efficacy and uptake of ART leads to the evolution of higher SPVL. In any given scenario, the value of SPVL that we predict to evolve is the evolutionarily stable phenotype (V*; solid lines). This optimized trait represents a compromise between the different values of SPVL that maximize transmission from untreated versus treated hosts. As one host type becomes more common, it comes to represent a greater potential source of HIV transmission and weighs more heavily on the optimization of SPVL. The relative proportions of untreated and treated infections are therefore integral to virulence evolution in this system, which is reflected by strong selection for increasing SPVL as the rate of drug uptake increases. However, when drugs are very highly effective (*r*_*T*_ ≥ 2), endemic equilibrium is only evolutionarily stable when drug uptake is very low, because no SPVL phenotype can evolve to maintain transmission in such well‐treated populations. While this plot shows that increasing the efficacy of ART leads to the evolution of higher SPVL, this trend reverses at very high efficacies, suggesting that intermediate leakiness selects for highest SPVL (see Appendix S4.2). Using a model that relaxes the assumption of constant population size, we find no difference in predicted evolutionary outcomes (see Appendix S4.4).

Pre‐exposure prophylaxis prevents infection without affecting the relative fitness of mutant viruses (see Equation S2.6), so in the absence of ART, PrEP bears no consequence on the evolution of SPVL. In our model, when ART is also available, the use of PrEP moves hosts into the treated class immediately upon infection. This acts to increase the proportion of infections exposed to ART, causing evolutionarily stable SPVL to become increasingly weighted by the transmission trade‐off in treated hosts. In this indirect way, the use of PrEP selects for higher SPVL (Figure 2). As PrEP and ART both affect the proportion of infections that become treated, viral evolution depends on the relative uptake rates of these two interventions. When PrEP uptake is low, the uptake rate of ART drives the proportion of hosts that are treated and hence the value of SPVL that evolves. Conversely, PrEP drives the evolution of SPVL when its uptake rate is high (darker green lines), as most infections become treated regardless of the ART uptake rate. This evolutionary influence of PrEP is most evident when its efficacy is low (*r*_*P*_ ≥ 0.2), because more effective PrEP often leads to situations where no value of SPVL is evolutionarily stable, in which case the virus always goes extinct. In Appendix S4.3, we further explore the evolutionary consequences of more effective PrEP and find that increasing PrEP efficacy tends to reduce selection for higher SPVL.

**Figure 3:**
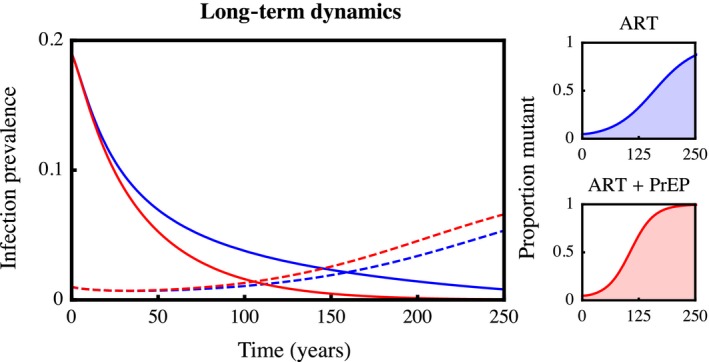
The prevalence of hosts with medium SPVL infections (solid lines) declines quickly in response to interventions, while high SPVL infections (dashed lines) remain relatively static over the short term and gradually increase in prevalence over the long term. The use of both ART and PrEP (red lines) leads to faster initial declines in the medium SPVL resident than when only ART is used (blue lines). However, PrEP also favours the transmission of high SPVL infections. Here, interventions have low efficacy (*r*_*T*_ = 1, *r*_*P*_ = 0.2) and relatively low uptake rates (*f*_*T*_ = 0.2, *f*_*P*_ = 0.01). Initially, 95% of infections have medium SPVL (*V* = 10^4.58^) and 5% have high SPVL (*V* = 10^5.5^)

While our evolutionary analyses provide no insight on the timescale of evolution towards higher virulence, we sought to address this question by running simulations of a medium SPVL resident “strain” competing with a high SPVL mutant, and circulating in a host population under different treatment regimes (Figure 3). We find that the introduction of ART leads to steep declines in medium SPVL infections, while the proportion of the host population harbouring high SPVL infections remains relatively static over the first several decades. Importantly, over longer timescales, high SPVL strains dominate and generate gradual rebounds in infection prevalence. Although the use of PrEP leads to faster declines in the prevalence of the medium SPVL resident, it also accelerates the transmission of the more virulent mutant over the long term. We also show in Appendix S4.5 how the relative proportion of resident and mutant infections varies with ART parameters, and in particular how intermediate leakiness facilitates the spread of high SPVL infections. However, we stress that the results of this strain competition model should not be interpreted as finely calibrated predictions, because they do not reflect the complexities and uncertainties that characterize real HIV epidemics.

**Figure 4:**
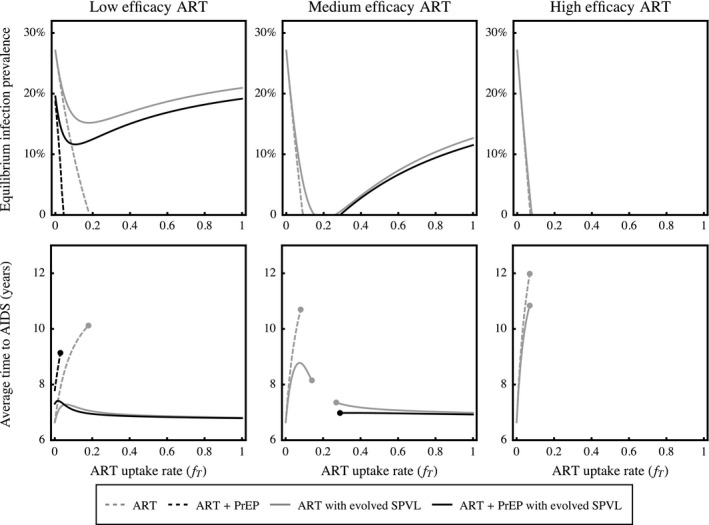
Epidemiological and clinical consequences of SPVL evolution. Top row: increasing the ART uptake rate leads to lower equilibrium infection prevalence in the absence of evolution (dashed lines), but can drive counterintuitive increases in prevalence when SPVL evolves (solid lines). Despite the stronger evolutionary response of SPVL when interventions are combined, equilibrium prevalence is always higher when ART is used alone (grey lines) than when ART and PrEP are used together (black lines, *f*_*P*_ = 0.01). Bottom row: increasing the use of ART increases the average duration of infection in the absence of evolution, but negates these clinical gains when it causes higher SPVL to evolve. Further, the addition of PrEP is always beneficial in the absence of evolution, but can lead to infections that progress to AIDS more quickly on average when SPVL evolves. Note that, in both rows, infections do not persist in the host population where lines are missing. As in Figure 2, PrEP is assumed to be low efficacy (*r*_*P*_ = 0.2). In Appendix S4.3, we plot the consequences of evolution in response to more effective PrEP

To understand the net effects of ART and PrEP, we determined the epidemiological and clinical consequences of having evolved virus circulating in host populations where those interventions were available. First, the evolution of SPVL allows endemic HIV infection to persist where it would otherwise be eliminated by interventions (see Fig. S4.7). More generally, equilibrium infection prevalences are always higher in populations exposed to evolved virus (dashed versus solid lines in Figure 4, top row), as higher SPVL leads to increased rates of transmission in treated populations. Surprisingly, when ART has low efficacy and its rate of uptake exceeds ~0.2, further increasing the availability of treatment leads to a higher prevalence of infection. This is because HIV evolves to maximize transmission from treated infections when they greatly outnumber untreated infections, and increasing the rate of ART uptake increases the frequency of viruses encountering the treated environment to which they are adapted. This explains why HIV infections with evolved SPVL sometimes persist in populations with high uptake rates, despite being unable to persist at intermediate uptake rates. Although ART also has the potential to prolong infections, which could ultimately lead to increased prevalence when treated hosts remain infectious, we show in Figure 4 that increases in infection prevalence are not accompanied by longer infections in our model. Importantly, the addition of PrEP always reduces equilibrium infection prevalence compared to when ART is used alone, despite the higher levels of SPVL that evolve in response to combined interventions (compare grey vs. black lines in Figure 4, top row).

Given default parameter estimates, asymptomatic infections persist for approximately 6.7 years before progressing to AIDS (slightly lower than the ~6.9 years calculated in Fraser et al. (2007), due to our inclusion of a background mortality rate; see Appendix S4.1). In the absence of evolution, increasing the uptake of ART lengthens the average time to AIDS, as treated infections have longer durations due to reduced viral loads (dashed grey lines in Figure 4, bottom row). However, when SPVL evolves in response to ART (solid grey lines), these clinical benefits are rapidly diminished, and in some cases increasing uptake can lead to worse clinical outcomes due to stronger selection for high SPVL viruses. Untreated hosts bear the brunt of this evolutionary cost, as they always progress to AIDS more quickly when the uptake and/or efficacy of ART increases (not shown). While the use of PrEP offers additional clinical gains in

the absence of evolution, it can lead to worse outcomes for hosts when SPVL evolves, as the population average time to AIDS is slightly lower when PrEP is available (compare grey vs. black solid lines in Figure 4, bottom row). In general, the epidemiological benefits and clinical costs of PrEP scale with its uptake rate (not shown), such that epidemics tend towards very high virulence, but also towards extinction as the use of PrEP increases.

## 4 Discussion

It is believed that HIV‐1 has evolved intermediate virulence to maximize population‐level transmission, but there are conflicting predictions on how the use of antiretroviral drugs is expected to affect this evolution (Herbeck et al., 2016; Payne et al., 2014; Roberts et al., 2015). Across taxa, there is growing experimental and observational support for the prediction that imperfect drugs and vaccines can lead to the evolution of higher virulence (e.g., Barclay et al., 2012; Gandon & Day, 2008; Read et al., 2015; Schneider et al., 2012), and HIV‐1 conforms to the conditions necessary for this kind of evolution (Gandon et al., 2001): it is an obligate endoparasite; its virulence and transmission both increase with viral density; and its medical interventions imperfectly reduce viral replication and susceptibility to infection. Here, we used an invasion analysis to predict evolutionary end‐points of set‐point viral load (SPVL, a proxy measure for virulence). Our approach contrasts with a recent individual‐based simulation study, which predicts the transient evolutionary dynamics of HIV virulence in response to ART (Herbeck et al., 2016), although we recapitulate their principal finding that fully suppressive antiretroviral therapy (ART) favours the transmission of higher SPVL strains under a test and treat policy. We further found that much higher SPVL is expected to evolve when ART is imperfect, or “leaky.” This evolution can allow the persistence of highly virulent HIV infections in conditions where drugs would otherwise eliminate the virus from a host population. In addition, preventive HIV drugs (pre‐exposure prophylaxis, or PrEP) reduce the prevalence of HIV in our model, but exacerbate the evolution of SPVL in response to ART. Counterintuitively, we show that when SPVL is allowed to evolve, higher uptake rates of ART or PrEP can be accompanied by higher HIV infection prevalence and worse clinical outcomes. While an untreated individual may fare worse if infected with a high SPVL strain, at the population level imperfect interventions always reduce HIV prevalence and almost always lengthen average time to AIDS compared to when antiretroviral drugs are altogether unavailable. Finally, we stress that increasing the efficacies of ART and PrEP (e.g., improving adherence) is shown to be an effective means of preventing higher SPVL from evolving.

Antiretroviral therapy has turned HIV infection from a death sentence into a manageable, lifelong condition (Ray, Logan, & Sterne, 2011), but only 40% of infections are believed to be treated (UNAIDS, 2014). While this suggests that the average rate of ART uptake is relatively low, access to treatment continues to improve in populations worldwide, entailing significant reductions in HIV‐1 incidence and AIDS‐related mortality (UNAIDS, 2014). However, consistent with contemporary work (Herbeck et al., 2016), our results show that increasing the uptake of ART may have unforeseen evolutionary consequences. Although we predict modest evolution in SPVL when drug uptake is low, evolutionarily optimal SPVL is expected to increase by orders of magnitude if leaky ART becomes very highly used. Intriguingly, average SPVL has increased in several European countries since the rollout of ART (Dorrucci et al., 2007; Gras et al., 2009; Müller et al., 2009; Potard et al., 2009). Although the causes of this evolution are unknown, our results suggest that the use of antiretroviral drugs may play a role in SPVL evolution in such highly treated populations.

Previous models of virulence evolution in response to ART have assumed that treated hosts do not transmit HIV (Herbeck et al., 2016; Payne et al., 2014; Roberts et al., 2015). In ideal conditions, infections undergoing ART are virologically suppressed and the risk of sexual transmission is negligible (Vernazza, Hirschel, Bernasconi, & Flepp, 2008). However, viral load is not suppressed in one quarter of treated infections worldwide (UNAIDS, 2014), and ART has been shown to reduce average HIV‐1 transmission risk by 42%–92% (reviewed in Attia, Egger, Müller, Zwahlen, & Low, 2009; Anglemyer et al., 2013 and Baggaley et al., 2013). Furthermore, Ratmann et al. (2016) recently found that 6% of infections in the Netherlands were transmitted from treated hosts. Regardless of its causes, this observed leakiness in ART is likely to elicit an evolutionary response in HIV if some treated infections are more likely to transmit than others because of viral traits. A further assumption of some previous work (Payne et al., 2014; Roberts et al., 2015) is that less virulent infections are less likely to receive treatment. Historically, the initiation of ART was delayed until the onset of AIDS (i.e., when CD4^+^ cell density declines below 200 cells/mm^3^; WHO 2010). As infections with high SPVL progress to AIDS more quickly, this treatment strategy disproportionately truncates the transmission window of infections with high SPVL, generating a transmission advantage for low SPVL infections. Administering treatment to some hosts over others is no longer standard practice, and contemporary test and treat policies encourage immediate initiation of ART upon HIV diagnosis (WHO, 2015), which obviates this mechanism of selection. However, universal treatment is not always possible, and there are likely to be treatment biases in some settings. For example, in Botswana, where Payne et al. (2014) observed the evolution of lower SPVL in comparison with neighbouring South Africa, prevention projects explicitly target ART towards those with the highest viral loads (Cohen et al., 2013).

Even when virulence evolves, our results suggest that increasing the use of PrEP has important epidemiological benefits. Indeed, infection prevalence is always lower in populations that use PrEP in addition to ART, even though combining these interventions leads to the evolution of higher SPVL. While this is good news from a public health perspective, evolution in response to ART and PrEP is accompanied by reduced clinical benefits of these drugs. In particular, virulence evolution in response to PrEP leads to infections that progress to AIDS more quickly than when ART is used on its own. Weighing the predicted epidemiological benefits of PrEP with these potential clinical costs is tricky. We note that when ART has low efficacy, virulence evolution leads to average infection durations that are effectively no different than baseline durations in untreated populations, regardless of whether the intervention is ART or combined ART and PrEP. Although interventions appear to neither help nor hinder clinical outcomes in these scenarios, we note that average infection duration is heavily weighted by treated hosts, who constitute the majority of infections when drug uptake is high, while untreated hosts experience a much faster time to AIDS when infected with evolved virus. This may be the most worrying risk we have uncovered. If imperfect interventions are sufficiently accessible, then, despite SPVL evolution, the epidemiological gains of leaky ART and PrEP may outweigh the degradation of clinical outcomes. However, HIV‐1 virulence evolution may have profound consequences if evolved viruses are introduced into populations where interventions are not available, or if drug resistance evolution reduces the efficacy of ART and thus removes the damper on the effects of higher SPVL.

We simplified the transmission dynamics of HIV in our model to make broad evolutionary and epidemiological predictions. First, we assumed no heterogeneity in hosts aside from their use of interventions. Second, apart from the constraints imposed by the virulence–transmission trade‐off, we assumed no limits to the evolution of SPVL. Although large variation in SPVL is observed, there are likely biological constraints such as host cell availability that preclude the evolution of extremely high SPVL. However, highly virulent HIV genotypes exist, and the full range of values of evolutionary stable SPVL predicted by our results has been observed in host populations (Fraser et al., 2007; de Wolf et al., 1997). Third, we simplified HIV‐1 infection as being wholly represented by the stable viral loads of asymptomatic infection. Although viral load and transmission rate surge during primary HIV‐1 infection (reviewed in Boily et al., 2009), the relative contribution of transmission during this brief period is contested. A recent study found that less transmission occurs during primary infection than previously thought (Bellan et al., 2015), and a review suggests that early infection may be less likely to play a significant role in populations where HIV is endemic (Miller, Rosenberg, Rutstein, & Powers, 2010). We do not expect the inclusion of primary HIV‐1 infection to qualitatively change our findings. Fourth, we assumed that viral suppression scales with baseline SPVL, such that infections with high SPVL remain more infectious than those with low SPVL when drugs are used. It is not known the extent to which this is true, but there are a number of observations that support this assumption. In particular, infections with higher SPVL have been shown to retain a higher viral load when treated (Maldarelli et al., 2007; Marconi et al., 2011; Wright et al., 2014), require a longer duration of treatment to achieve virologic suppression (Manegold et al., 2004; Matthews et al., 2002; Mugavero et al., 2012; Paredes et al., 2000; Patel, Mario, Thorne, & Newell, 2007; Phillips et al., 2001; Rizzardi et al., 2000), be less likely to achieve complete virologic suppression (Bratt et al., 1998; Chaisson, Keruly, & Moore, 2000; Crawford, Sanderson, & Thornton, 2014; Knobel et al., 2001; Paredes et al., 2000) and be more likely to experience virologic failure (i.e., viral rebound despite adherence to ART; Egger et al., 2002; van Leth et al., 2005). Furthermore, SPVL rebounds rapidly when treatment is stopped (Davey et al., 1999; García et al., 1999; Ruiz et al., 2000) and typically to pretreatment levels (Hamlyn et al., 2012; Hatano et al., 2000; Oxenius et al., 2002). Prolonged breaks in adherence may thus represent islands of infectivity where host infectiousness relates positively to pretreatment SPVL. Finally, viral “blips” (brief and intermittent periods of detectable viral load despite adherence to ART) are observed more often in hosts with high baseline SPVL (Havlir et al., 2001; Leierer et al., 2015), and infections with large or frequent blips are more likely to experience virologic failure (Easterbrook et al., 2002; Grennan et al., 2012; Laprise, De Pokomandy, Baril, Dufresne, & Trottier, 2013) and achieve higher viral rebound upon treatment interruption (Castro et al., 2013). These observations span drug types, host populations and viral clades, but collectively support the intuitive assumption that infections with high SPVL are more infectious than those with low SPVL when imperfectly treated.

The HIV‐1 pandemic is a dynamic assemblage of host populations infected with diverse viral subtypes and exposed to shifting public health interventions. Not surprisingly, then, there are conflicting reports of the strength and direction of HIV‐1 virulence evolution and the mechanisms that drive it (Blanquart et al., 2016; Fraser et al., 2014; Herbeck et al., 2012, 2016; Payne et al., 2014). Nevertheless, there is evidence that average SPVL has increased in several highly treated populations since the start of the pandemic (reviewed in Herbeck et al., 2012), and Herbeck et al. (2016) show that ART can select for higher virulence by shortening the transmission window in infected hosts. Building on this finding, our work demonstrates that leakiness in HIV treatment also selects for higher SPVL, reducing clinical advantages of treatment and making the virus more difficult to eliminate from host populations. We further predict that PrEP exacerbates the evolutionary response of SPVL, while reducing infection prevalence. These results suggest that imperfect efficacy in HIV interventions may have under‐appreciated consequences for the evolution of virulence, but that these consequences can be avoided if high efficacies of ART and PrEP are assured as these interventions continue to become more available. Additionally, integrated control strategies that combine drugs with other approaches (e.g., condoms) should remain at the forefront of prevention to mitigate leakiness in ART and PrEP. Finally, as access to ART and PrEP continues to improve, monitoring both SPVL evolution and drug efficacies should remain high priorities to ensure the long‐term benefits of these life‐saving interventions.

## Conflict of interests

The authors declare no conflict of interests.

## Data archiving

Data available from the Dryad Digital Repository: https://doi.org/10.5061/dryad.26767
