# Supplementary material for: Modelling the evolution of HIV‐1 virulence in response to imperfect therapy and prophylaxis
Source: Evol Appl. 2017 Feb 11;10(3):297–309. doi: 10.1111/eva.12458 (PMC5322411; doi:10.1111/eva.12458)
Supplement: Supplementary file 1 [file EVA-10-297-s001.pdf]

Appendix S1 Equilibrium analyses

The basic model of HIV transmission presented in the main text (equations 1 to 4) has one disease-free equilibrium, DFE,

$$\hat{S}_{DFE} = \frac{\mu}{\mu + f_P} \tag{S1.1}$$
$$\hat{P}_{DFE} = \frac{f_P}{\mu + f_P} \tag{S1.2}$$
$$\hat{I}_{DFE} = 0 \tag{S1.3}$$
$$\hat{T}_{DFE} = 0 \tag{S1.4}$$

where circumflexes denote equilibrium host frequencies. When PrEP is not used, this reduces to $\{\hat{S}_{DFE} = 1, \hat{P}_{DFE} = 0, \hat{I}_{DFE} = 0, \hat{T}_{DFE} = 0\}$.

We also find an endemic equilibrium, EE (with a non-zero equilibrium proportion of infected hosts), which depends on the interventions used in the population. When neither PrEP nor ART are available ($f_T = f_P = 0$),

$$\hat{S}_{EE_1} = \frac{\mu + \alpha_I}{\beta_I} \tag{S1.5}$$
$$\hat{P}_{EE_1} = 0 \tag{S1.6}$$
$$\hat{I}_{EE_1} = 1 - \frac{\mu + \alpha_I}{\beta_I} \tag{S1.7}$$
$$\hat{T}_{EE_1} = 0 \tag{S1.8}$$

where, assuming a low background rate of mortality, the equilibrium prevalence of infection is determined largely by the trade-off between transmission rate and disease progression. When imperfect ART is introduced into the population ($f_T > 0$), then

$$\hat{S}_{EE_2} = \frac{\mu + \alpha_I + f_T}{\beta_I + \frac{f_T \beta_T}{\mu + \alpha_T}} \tag{S1.9}$$
$$\hat{P}_{EE_2} = 0 \tag{S1.10}$$
$$\hat{I}_{EE_2} = \frac{\frac{(\mu+\alpha_T)(\beta_I-\alpha_I)+f_T(\beta_T-\alpha_T)}{\mu+\alpha_T+f_T} - \mu}{\beta_I + \frac{f_T\beta_T}{\mu+\alpha_T}} \tag{S1.11}$$
$$\hat{T}_{EE_2} = \frac{f_T}{\mu + \alpha_T}\hat{I}_{EE_2} \tag{S1.12}$$

where increasing the rate of ART uptake (f_T) leads to an increasing proportion of treated relative to untreated infections. ART eliminates infection from the host population when

$$f_T \geq \frac{(\beta_I - \mu - \alpha_I)(\mu + \alpha_T)}{\mu + \alpha_T - \beta_T} \tag{S1.13}$$

and so the uptake rate of drugs that achieves disease eradication depends, as would be expected, on the relative efficacy of ART (r_T) and the values of α_T and β_T that result.

When imperfect PrEP ($0 < r_P < 1$) is introduced ($f_P > 0$) in the absence of ART ($f_T = 0$), analytic equilibrium

expressions are intractable (i.e., few simple inferences can be drawn from them) and so are not shown. However, when PrEP always prevents infection ($r_P = 1$), we find,

$$\hat{S}_{EE_3} = \frac{\mu + \alpha_I}{\beta_I} \tag{S1.14}$$

$$\hat{P}_{EE_3} = \frac{f_P(\mu + \alpha_I)}{\mu\beta_I} \tag{S1.15}$$

$$\hat{I}_{EE_3} = \frac{\mu(\beta_I - f_P - \mu) - \alpha_I(f_P + \mu)}{\mu\beta_I} \tag{S1.16}$$

$$\hat{T}_{EE_3} = 0 \tag{S1.17}$$

where, predictably, increasing the rate of PrEP uptake leads to fewer infections, and endemic infection is eliminated when

$$f_P \geq \frac{\mu(\beta_I - \mu - \alpha_I)}{\mu + \alpha_I}. \tag{S1.18}$$

Comparing this to equation S1.13 and considering the case that ART is also perfectly effective ($r_T = \infty$, and therefore $V_T = \beta_T = 0$), we found that infection is eliminated for lower values of f_P than f_T. Thus, all else being equal, preventing infection in susceptible hosts is a more effective way to control the spread of the virus than preventing transmission from infected individuals.

When both ART and PrEP are used simultaneously ($f_T, f_P > 0$), analytic equilibrium expressions are only solvable when drugs work with perfect efficacy:

$$\hat{S}_{EE_4} = \frac{\mu + \alpha_I + f_T}{\beta_I} \tag{S1.19}$$

$$\hat{P}_{EE_4} = \frac{f_P(\mu + \alpha_I + f_T)}{\mu\beta_I} \tag{S1.20}$$

$$\hat{I}_{EE_4} = \frac{\mu - \frac{(\mu + f_P)(\mu + \alpha_I + f_T)}{\beta_I}}{\mu + f_T} \tag{S1.21}$$

$$\hat{T}_{EE_4} = \frac{f_T - \frac{f_T(\mu + f_P)(\mu + \alpha_I + f_T)}{\mu\beta_I}}{\mu + f_T}. \tag{S1.22}$$

In this case, endemic infection is eliminated when

$$f_T \geq \mu\Big(\frac{\beta_I}{\mu + f_P} - 1\Big) - \alpha_I \tag{S1.23}$$

or

$$f_P \geq \mu\Big(\frac{\beta_I}{\mu + \alpha_I + f_T} - 1\Big). \tag{S1.24}$$

Finally, because equilibria were not always analytically solvable, we used numerical simulations to predict the effects of imperfect PrEP and ART on equilibrium infection prevalence. Host frequencies only converge to equilibrium values that are locally asymptotically stable, and so a population is only infected when its EE is stable. Equilibrium stability was found by evaluating the local stability matrix (the Jacobian matrix evaluated at the equilibrium). All endemic equilibria that we report were found to be locally asymptotically stable.

Appendix S2 Invasion thresholds

From the evolutionary invasion analysis presented in the main text, we calculated invasion thresholds, i.e., the conditions under which a mutant virus is expected to invade. A rare mutant virus invades the resident population when its $R_0 > 1$. In the absence of interventions ($f_T = f_P = 0$), we recapitulate R_0 as in the classic SI model of parasite transmission (Otto and Day, 2008), and expect a mutant to invade when

$$\frac{\beta'_I \hat{S}}{\mu + \alpha'_I} > 1. \tag{S2.1}$$

Substituting $\hat{S}$ from equation S1.5, gives

$$\frac{\beta'_I}{\mu + \alpha'_I} > \frac{\beta_I}{\mu + \alpha_I} \tag{S2.2}$$

and so, intuitively, a mutant invades when it transmits to more hosts per infectious period than the resident.

The introduction of ART alters R_0, such that mutant invasion occurs when

$$\frac{(\beta'_I + \frac{f_T \beta'_T}{\mu + \alpha'_T})\hat{S}}{\mu + \alpha'_I + f_T} > 1. \tag{S2.3}$$

Substituting $\hat{S}$ from equation S1.9, gives

$$\frac{\beta'_I + f_T \frac{\beta'_T}{\mu + \alpha'_T}}{\mu + \alpha'_I + f_T} > \frac{\beta_I + f_T \frac{\beta_T}{\mu + \alpha_T}}{\mu + \alpha_I + f_T}. \tag{S2.4}$$

Now, the expressions for R_0 of both the mutant and resident represent the sum of the per-host type components of R_0. The first term in the numerator (on either side of the inequality) describes the infection of susceptible hosts by untreated hosts; the second term describes the infection of susceptible hosts by treated hosts, and accounts for the rate at which infections progress into the treated class.

In the absence of ART, the introduction of PrEP alters R_0 such that invasion occurs when

$$\frac{\beta'_I(\hat{S} + \eta_P \hat{P})}{\mu + \alpha'_I} > 1. \tag{S2.5}$$

Although expressions for $\hat{S}$ and $\hat{P}$ are complex and are not shown, when they are substituted into the above equation we find

$$\frac{\beta'_I}{\mu + \alpha'_I} > \frac{\beta_I}{\mu + \alpha_I}, \tag{S2.6}$$

which is the same invasion condition as when no interventions are used at all. Since neither the efficacy nor uptake rate of PrEP affect a mutant's invasion fitness, PrEP has no bearing on the evolution of V when it is the only intervention being used. This is analogous to the finding in Gandon et al. (2001) that anti-infection vaccines have no impact on virulence evolution in the absence of other forces (e.g., other interventions).

When imperfect PrEP and ART are used simultaneously, analytical expressions for equilibrium host frequencies were

not found, but a mutant invasion threshold could still be partially derived, and is given by

$$\frac{(\beta_I' + \frac{f_T \beta_T'}{\mu+\alpha_T'})\hat{S}}{\mu + \alpha_I' + f_T} + \frac{\eta_P \beta_T' \hat{P}}{\mu + \alpha_T'} > 1. \quad \text{(S2.7)}$$

Again, this is interpreted as the sum of the per-host components of R_0, where the first term describes the infection of susceptible hosts, from any source (as above), and the second term describes the infection of hosts using PrEP.

Appendix S3 Numerical determination of evolutionary stability

In order to determine the evolutionary stability of SPVL fitness maxima, we calculated second derivative conditions of the respective selection gradients. An example of this is plotted in Figure S3.1, where we show that the second derivative condition (inequality 24 in the main text) is always met when ART has low efficacy, but has a zero value and is not met when ART has high efficacy and a high rate of uptake. When the second derivative condition is positive, evolutionary branching occurs, i.e. multiple stable trait values can evolve from one monomorphic parasite population. This is sometimes observed in models of virulence evolution when different virulence traits evolve that are specialized to different host types (Otto and Day, 2008). However, despite the ecological differences between untreated and treated hosts, our model does not predict this result, even when treatment status is always determined prior to infection (i.e., $f_T = 0, f_P > 0$).

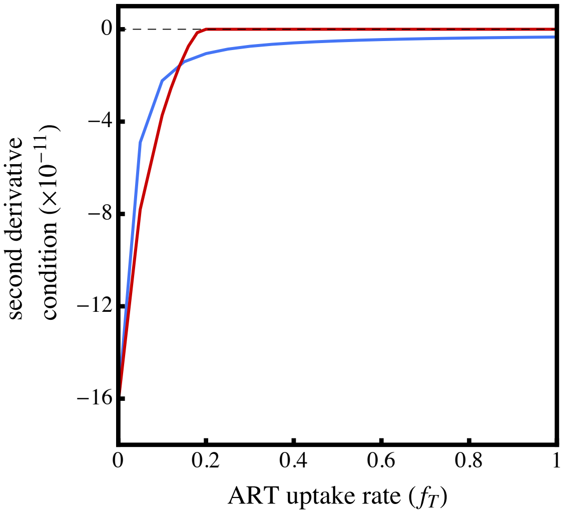

Figure S3.1: When the second derivative condition of the selection gradient is negative, the corresponding root is convergence stable, and hence evolutionarily stable, and this is the trait value expected to evolve over time. When ART has low efficacy ($r_T = 1$, blue line), the value of SPVL that maximizes transmission is always convergence stable and evolves in our model population. However, when ART is highly effective ($r_T = 2$, red line) and its uptake rate $f_T > 0.22$, the second derivative condition is zero, and so the value of SPVL that maximizes transmission is not convergence stable, and does not evolve in this model (compare with corresponding lines in Figure 4 in the main text).

Appendix S4 Supplementary Results

S4.1 Evolutionary consequences of background mortality

Fraser et al. (2007) estimated that the SPVL trade-off in HIV-1 maximizes transmission potential when $V = 10^{4.52}$ virions/mL. However, this optimal trait value was calculated using 'transmission potential' (i.e., transmission rate multiplied by the duration of infection) as a proxy for fitness, which does not include alternative sources of mortality and demographic feedbacks. Background mortality rate is a standard term in epidemiological models, and increasing this rate (μ) selects for an increasing rate of host exploitation, which leads to the evolution of higher virulence (Anderson and May, 1981; Williams and Day, 2001). This is recapitulated in our model, where R_0 is maximized at $V = 10^{4.52}$ when $\mu = 0$, but at increasing V as μ increases (Figure S4.1). This means that the evolutionary stable viral load that we calculate in an untreated population is slightly higher than that calculated by Fraser et al. (2007). However, since our rate of background mortality is low ($\mu = 0.02$) and consistent across all host types, the inclusion of this parameter does not qualitatively influence our results.

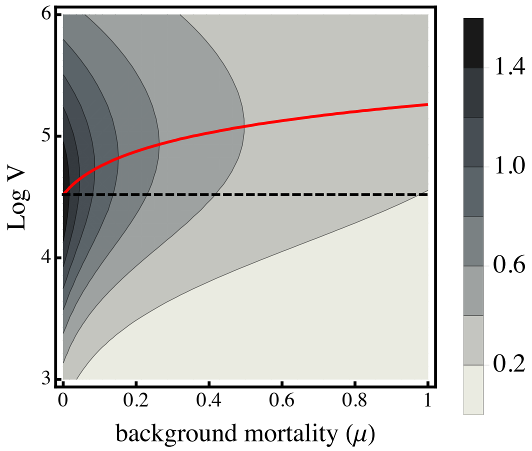

Figure S4.1: Contour plot showing the viral reproduction ratio (R_0) as a function of $\log_{10}$ SPVL (V) and the background mortality rate (μ). The value of V that maximizes 'transmission potential' was calculated by Fraser et al. (2007) as $V = 10^{4.52}$ (black dashed line). However, the value of V that maximizes lifetime HIV transmission increases with μ (red solid line), and so the inclusion of a background mortality rate leads to the evolution of higher SPVL.

S4.2 Evolutionary consequences of ART efficacy

Herbeck et al. (2016) have shown that the length of time until ART initiation (i.e., the inverse of the rate of ART uptake) affects the evolution of SPVL under a test and treat policy, because treatment initiation terminates the transmission window at the same time in all hosts regardless of SPVL, offering a transmission advantage to the hosts that are most infectious during untreated infection. Additionally, we show that when drugs have imperfect efficacy, the SPVL tradeoff is optimized differently in untreated and treated infections, and the value of SPVL that evolves depends on the relative contribution to transmission of these two host types. Further, we show in the main text that, for any given rate of ART uptake, more effective ART tends to select for higher SPVL (Figure 2). However, this is not always the case, because untreated hosts continue to account for the majority of transmission when ART is extremely effective, even if the majority of hosts are treated. In this way, qualitatively intermediate levels of leakiness select for the highest levels

of SPVL, but the specific levels of ART efficacy that maximize SPVL depend on the rate of treatment uptake (Figure S4.2).

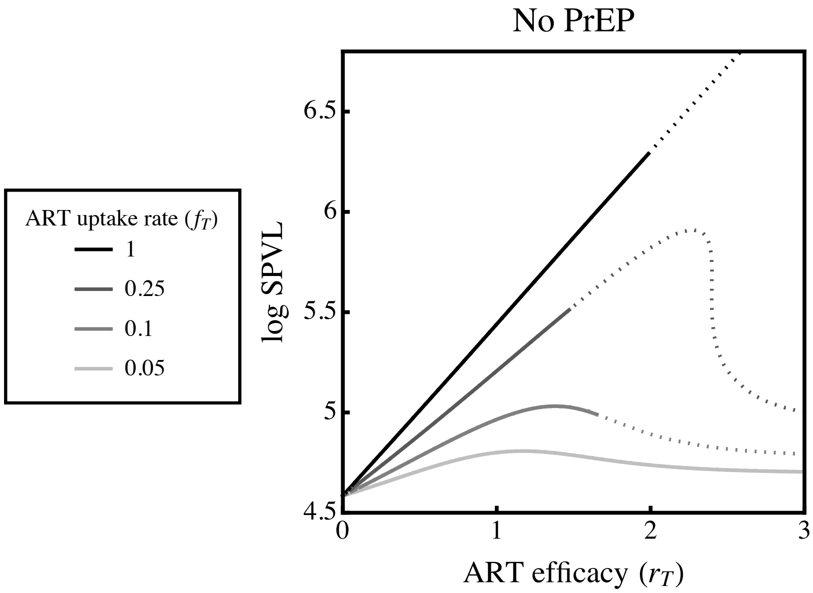

Figure S4.2: The levels of SPVL that maximize transmission depend on both ART efficacy and uptake. Evolutionarily stable SPVL (V^*, solid lines) decreases over high values of ART efficacy when drug uptake rates are low. This is because, when drugs approach high efficacy but most infections are untreated, SPVL evolves to lower levels that maximize transmission from untreated hosts. Although this trend continues at higher ART uptake rates, the levels of SPVL that maximize transmission are much greater but are often evolutionarily unstable (dotted lines).

S4.3 Consequences of more effective PrEP

In the main text, we explored the evolutionary consequences of low efficacy PrEP ($r_P = 0.2$). Here, we extend our analysis to include medium efficacy PrEP ($r_P = 0.5$). As we were unable to find complete analytical solutions for R_0 when leaky PrEP is used, we estimated the levels of SPVL that maximize transmission when the host population is at disease-free equilibrium by substituting equilibria S1.1 and S1.2 into equation S2.7 (Figure S4.3). No hosts on PrEP become infected when it works with perfect efficacy, and so the level of SPVL that maximizes transmission is not affected by PrEP uptake. However, as the efficacy of PrEP decreases, higher values of SPVL maximize transmission because more hosts on PrEP become infected and enter the treated class.

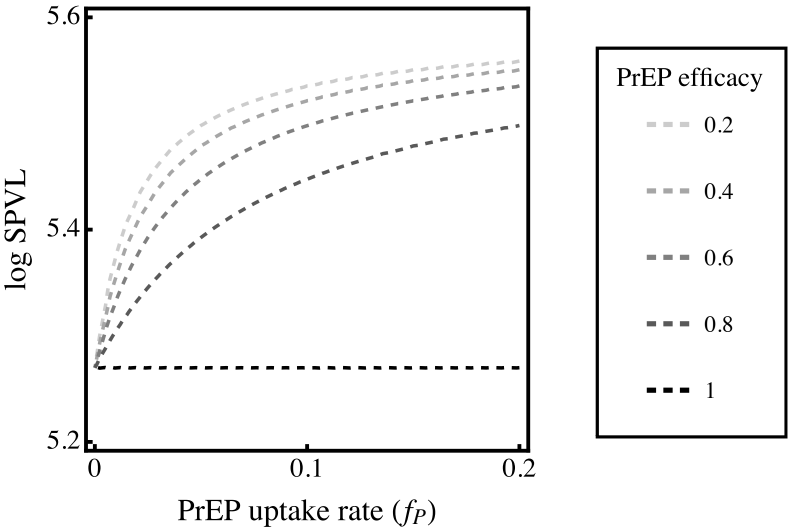

Figure S4.3: When PrEP is perfectly effective (black lines), increasing its uptake rate has no effect on the level of SPVL that maximizes transmission (dashed lines). As PrEP becomes less effective (lighter grey), more hosts become infected while on PrEP, and so the proportion of treated infections increases and higher SPVL is favoured. Here, ART has low efficacy ($r_T = 1$) and intermediate uptake ($f_T = 0.33$).

We also extended our analysis to explore how SPVL evolution affects epidemiological outcomes when PrEP has intermediate efficacy ($r_P = 0.5$). We find that higher efficacy PrEP leads to eradication of HIV over a broader range of ART uptake rates, and that HIV can only be evolutionarily stable when ART has low efficacy. As expected, higher efficacy PrEP reduces equilibrium infection prevalence (Figure S4.4, top row), but also entails minor reductions in the average duration of HIV infection (Figure S4.4, bottom row). When PrEP is highly effective ($r_P = 0.8$), epidemics almost never persist at equilibrium, whether or not SPVL evolves (not shown).

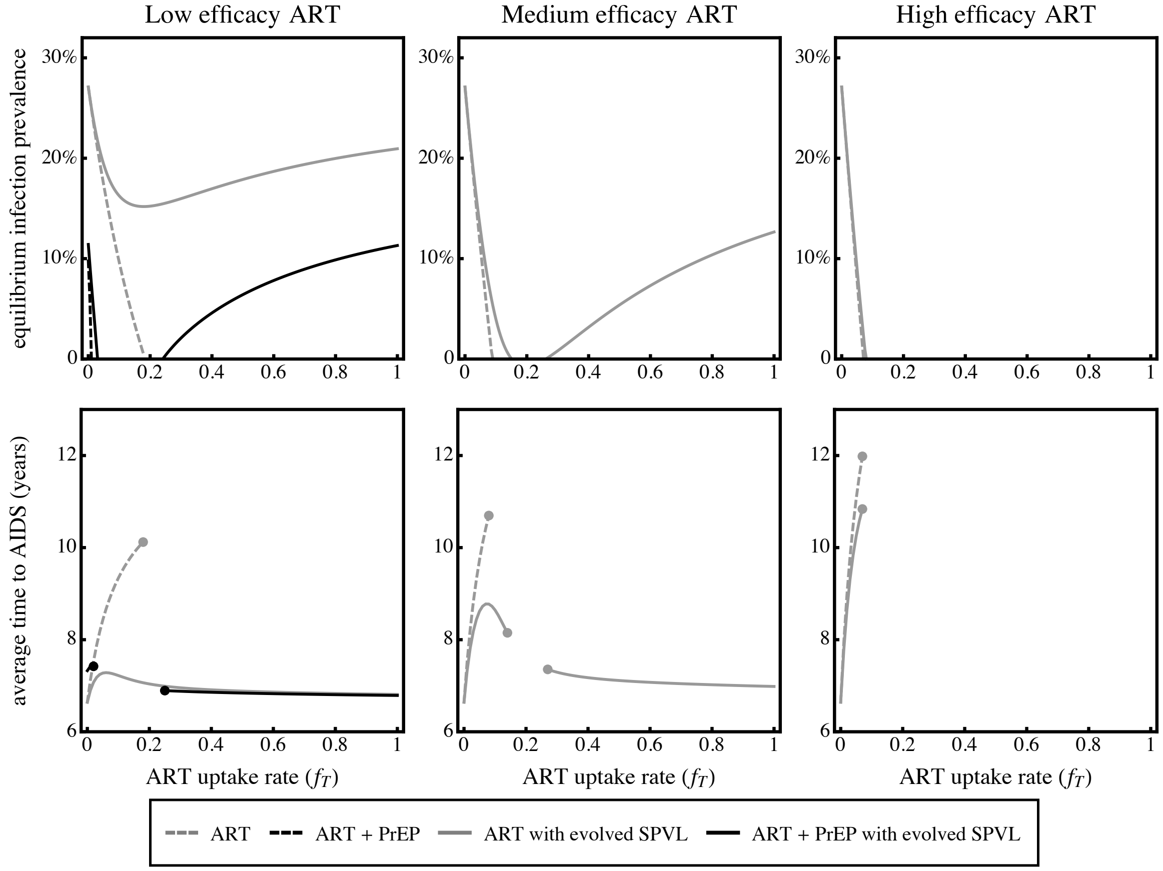

Figure S4.4: Epidemiological and clinical consequences of medium efficacy PrEP (here, $r_P = 0.5$) are qualitatively similar to when PrEP has low efficacy ($r_P = 0.2$, as in Figure 4 in the main text), but epidemics only persist when ART has low efficacy ($r_T = 1$, left column). Where HIV evolves in response to PrEP and hence persists in the population (solid lines), infection prevalence is lower when PrEP has medium efficacy than low efficacy, but the average duration of infection is slightly shorter. As in the main text, PrEP uptake $f_P = 0.01$.

S4.4 Evolutionary consequences of constant population size

We relaxed the assumption of constant population size by rerunning our model with a constant birth rate (θ_C). The equilibrium number of infected hosts increases with θ_C, but the equilibrium prevalence of infection is unchanged, because θ_C drops out of prevalence expressions in models of frequency-dependent parasite transmission. Furthermore, θ_C does not appear in the next-generation matrix in this modified model, and so we found that evolutionarily stable SPVL is identical in models with constant and non-constant birth rates.

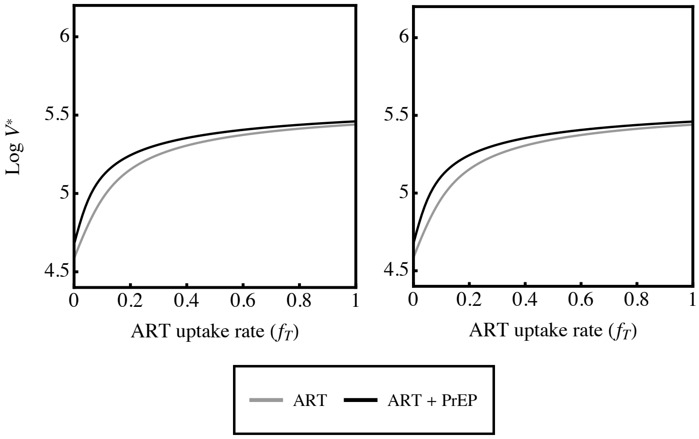

Figure S4.5: The choice of birth rate does not affect the evolutionary stability of SPVL (solid lines). **Left:** Population size is held constant at $N = 1$. **Right:** Population size varies due to a constant birth rate (here, $\theta = 20$).

S4.5 How interventions and evolution affect HIV persistence and extinction

In Figure S4.6, we show that the proportion of infections caused by a high SPVL strain jumps from 5% to over 50% in just 50 years when ART has low efficacy ($r_T \approx 1$). The high SPVL mutant is most common when ART has intermediate efficacy, suggesting that preventing leakiness in treatment by maximizing ART efficacy may be a means of preventing virulence evolution in HIV. The parameters used here are the ones used used in Figure 3 in the main text.

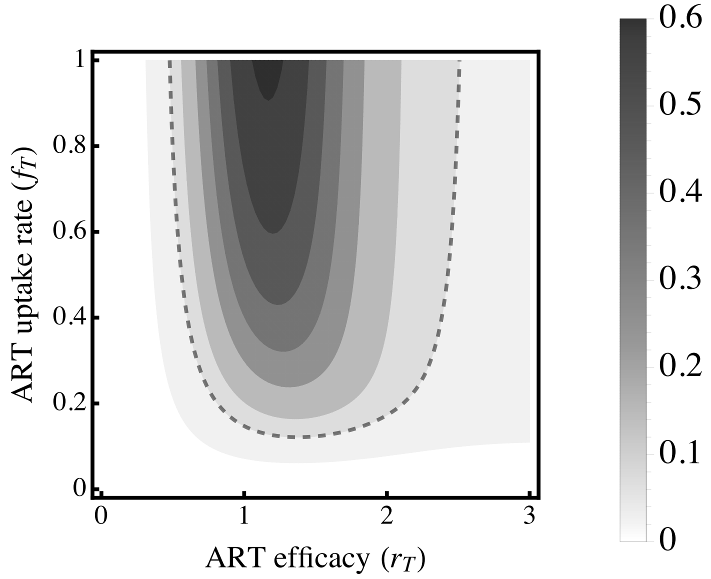

Figure S4.6: Contours depict the proportion of infections caused by a high SPVL mutant 50 years after the introduction of ART. In this example, the greatest proportion of infections are high SPVL (darkest grey) when ART has low to intermediate efficacy ($1 < r_T < 1.5$) and high rate of uptake. Our simulations assumed that prior to ART ($t = 0$), 20% of hosts were infected, and 5% of those infections were caused by high-SPVL mutants ($10^{5.5}$), while the remaining 95% of infections had medium SPVL ($10^{4.5}$). The dashed black contour indicates 5%, and therefore darker grey regions inside this line indicate that the mutant has increased in prevalence proportional to the wild-type over time.

In Figure S4.7, we explore the parameter space in which interventions lead to eradication of disease, either ignoring or accounting for viral evolution. These plots show where viral evolution allows HIV to persist in conditions where interventions would otherwise drive the virus to extinction (light grey shaded regions). They also delineate the combinations of drug parameters that are predicted to be robust to viral evolution (dark grey shaded regions).

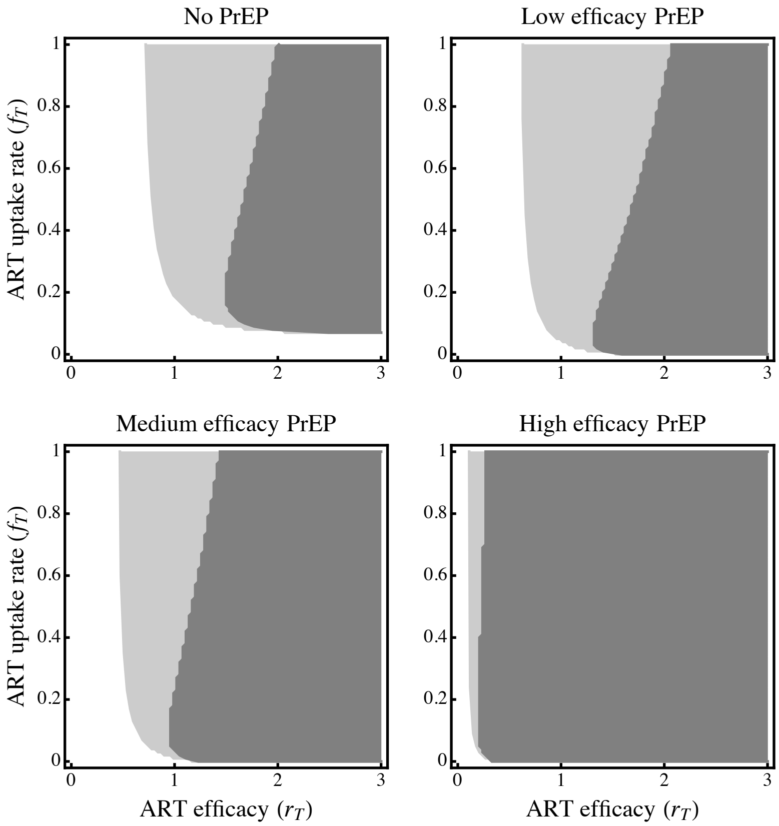

Figure S4.7: Interventions can drive HIV to extinction, but this depends on whether or not SPVL evolves. Dark grey areas show the parameter space in which interventions lead to eradication of HIV, even in the face of viral evolution. When SPVL does not evolve, interventions can additionally lead to eradication in the light grey regions. Here, the PrEP uptake rate is low ($f_P = 0.01$).

We predicted the duration of time over which HIV interventions lead to the elimination of infection from the host population. In doing so, we assume that SPVL does not evolve. We first infect a naïve population with default virus ($V = 38,465$) and let it reach its endemic equilibrium

$$\{\hat{S}_{EE}, \hat{P}_{EE}, \hat{I}_{EE}, \hat{T}_{EE}\} = \{0.729, 0, 0.271, 0\}. \tag{S4.1}$$

We then introduce an intervention at time zero. In the case that the endemic equilibrium is destabilized by the intervention, we determine how long it takes for the virus to go extinct by plotting the population dynamics over time and determining when no more transmission occurs, or when

$$\hat{I} + \hat{T} \approx 0. \tag{S4.2}$$

Disease eradication happens more quickly as the efficacy and uptake rate of ART increase, but in optimistic interven-

tion scenarios it can still take decades if not centuries for the virus to be eliminated (Figure S4.8, left). For example, when drugs are very effective and are initiated at a very high rate ($r_T = 3, f_T = 1$), it still takes an estimated 87 years for infection to be altogether eliminated from the population. Combination interventions lead to the most rapid disease elimination, and we show how increasing the uptake rates of PrEP and ART decreases the time until viral extinction (Figure S4.8, right). In a scenario of very high ART and PrEP uptake ($f_T = f_P = 1$), HIV is eliminated in approximately 76 years.

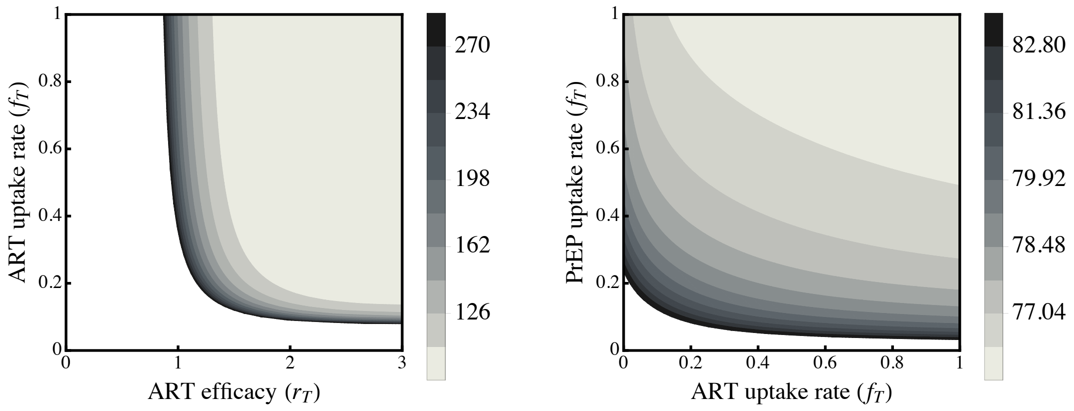

Figure S4.8: Contour plots showing the estimated number of years until HIV is eradicated with the use of ART (left) or ART and PrEP (right). As drugs become more potent and more widely used, HIV is eradicated more quickly. For the combined effect of ART and PrEP we assume low efficacy values ($r_P = 0.2, r_T = 1$).

References

Anderson, R. M. and May, R. M. (1981). The population dynamics of microparasites and their invertebrate hosts. *Philosophical Transactions of the Royal Society B*, 291:451–524.

Fraser, C., Hollingsworth, T. D., Chapman, R., de Wolf, F., and Hanage, W. P. (2007). Variation in HIV-1 set-point viral load: epidemiological analysis and an evolutionary hypothesis. *Proceedings of the National Academy of Sciences*, 104:17441–6.

Gandon, S., Mackinnon, M. J., Nee, S., and Read, A. F. (2001). Imperfect vaccines and the evolution of pathogen virulence. *Nature*, 414:751–756.

Herbeck, J. T., Mittler, J. E., Gottlieb, G. S., Goodreau, S., Murphy, J. T., Cori, A., Pickles, M., and Fraser, C. (2016). Evolution of HIV virulence in response to widespread scale up of antiretroviral therapy: a modeling study. *Virus Evolution*, 2:vew028.

Otto, S. and Day, T. (2008). *A Biologist's Guide to Mathematical Modeling in Ecology & Evolution*. Princeton University Press.

Williams, P. D. and Day, T. (2001). Interactions between sources of mortality and the evolution of parasite virulence. *Proceedings of the Royal Society B: Biological Sciences*, 268:2331–7.
